# Stendhal syndrome: Can art make you ill?

**DOI:** 10.1192/j.eurpsy.2021.852

**Published:** 2021-08-13

**Authors:** G. Marinho, J. Peta, J. Pereira, M. Marguilho

**Affiliations:** 1 Clinica 6, CHPL, Lisbon, Portugal; 2 Psychiatry, Centro Hospitalar Psiquiátrico de Lisboa, Lisboa, Portugal; 3 Clínica 5, Centro Hospitalar Psiquiátrico de Lisboa, Lisboa, Portugal

**Keywords:** Stendhal syndrome, travel syndromes, culture shock

## Abstract

**Introduction:**

A psychosomatic disorder, Stendhal Syndrome, causes tachycardia, dizziness, sweating, disorientation, fainting, and confusion when someone is looking at artwork with which he or she connects deeply emotionally. In 1817, a French author named Marie-Henri Beyle, whose pseudonym was Stendhal, described his experience visiting the Basilica of Santa Croce in Florence and feeling overwhelmed by all the beauty and rich history surrounding him. Over a century later, visitors to Florence continued to suffer from similar symptoms. In 1979, Dr. Graziella Magherini, Chief of Psychiatry at the Hospital of Santa Maria Nuova in Florence, observed more than 100 tourists who were hospitalized after looking at art in Florence and coined the term Stendhal Syndrome.

**Objectives:**

To review literature on Stendhal syndrome, a bizarre travel-related syndrome.

**Methods:**

PubMed and Google Scholar search using the keywords Stendhal syndrome, travel syndromes, culture shock

**Results:**

Victims are typically impressionable, single people between 26-40 years old, who are stressed by travel and may be struggling with jet lag. For art lovers, the thrill of arriving somewhere like Florence that gathers so much famous art is like meeting all your heroes at once. This strange aesthetic sickness is surely evidence of the special power of Renaissance art.

**Conclusions:**

Stendhal Syndrome does not currently appear in the DSM-5 (Diagnostic and Statistical Manual of Mental Disorders). Psychiatrists have however, documented the syndrome in medical journals and advise that tourists pace themselves in art museums and get enough rest in between viewings of Italy’s breathtaking, powerful masterpieces.

